# An Evaluation of the Accuracy and Precision of Ceramic Orthodontic Bracket Slot Dimensions Utilizing Micro-Computed Tomography (Micro-CT)

**DOI:** 10.3390/tomography9040109

**Published:** 2023-07-13

**Authors:** Antonio Garrett, Maryam A. Alghilan, Simon Ash, Mohammed Awawdeh, Parmjit Singh

**Affiliations:** 1 Faculty of Dentistry, BPP University, 32-34 Colmore Circus Queensway, Birmingham B4 6BN, UK; aacostareis@gmail.com; 2 Restorative and Prosthetic Dental Sciences Department, College of Dentistry, King Saud bin Abdulaziz University for Health Sciences (KSAU-HS), Riyadh 11426, Saudi Arabia; ghilanm@ksau-hs.edu.sa; 3 King Abdullah International Medical Research Center, Ministry of National Guard Health Affairs, Riyadh 11481, Saudi Arabia; 4 Ministry of the National Guard—Health Affairs, Riyadh 11426, Saudi Arabia; 5 College of Medicine & Dentistry, Ulster University, 32-34 Colmore Circus, Birmingham B4 6BN, UK; simon.maria@tiscali.co.uk; 6 Preventive Dental Science Department, College of Dentistry, King Saud bin Abdulaziz University for Health Sciences (KSAU-HS), Riyadh 11426, Saudi Arabia

**Keywords:** micro-CT, accuracy, brackets, orthodontics, torque, ceramics, precision

## Abstract

This study’s aim is to determine the accuracy and precision of the bracket slot height in MBT 0.022″ ceramic brackets. Five brackets from 11 different systems (n = 55) were scanned using micro-computed tomography (micro-CT). The slot height was measured at the face (external) and base (internal) of the slot. Data were analyzed using a One-Sample *t*-Test, and a Paired *t*-Test. The lowest external height was seen in OrthoCare Purity^®^ at 0.02264″ (2.9%), and the greatest in TOC Ghost Advanced^®^ at 0.02736″ (24.4%). The lowest internal height was seen in Forestadent Glam^®^ at 0.02020″ (−8.2%), and the greatest in TOC Ghost Advanced^®^ at 0.2547″ (15.8%). OrthoCare Purity^®^ measurements corresponded most closely with the expected measurements. TP InVu^®^ was found to be the most precise bracket for external height (range = 0.00043″) and American Orthodontics 20/40^®^ for internal height (range = 0.00028″). In assessing slot geometry, all brackets demonstrated a higher mean external slot height compared to the internal measurements at the base of the bracket. Orthodontic bracket slots are larger than expected and slot parallelism was not observed in any bracket brand tested. Similarly, slot dimensions are imprecise where two ‘identical’ brackets have different slot sizes. The clinician should, therefore, assume that play is most likely higher than expected.

## 1. Introduction

Contemporary orthodontic brackets have a built-in prescription that will enable three-dimensional tooth movements during a course of orthodontic treatment. The built-in prescription revolutionized fixed orthodontic appliances and significantly reduced the requirement for wire bending [1]. The forces that are intended to be produced by a pre-adjusted edgewise bracket are highly dependent on the intimate fit of the wire into the bracket slot and the greater the ‘play’ between these two, the more inefficient is the transmission of the bracket prescription to the tooth and supporting bone [2].

Modern ceramic orthodontic brackets are produced from aluminum oxide (alumina) particles. Mostly, these brackets are available in polycrystalline form, produced by ceramic injection molding, which fuses multiple aluminum oxide particles into a shape from which a bracket can be cut. They are also available in monocrystalline form, produced by the milling of a large single aluminum oxide crystal [3]. The manufacturing process of orthodontic brackets has been known to introduce discrepancies in the accuracy of the slot dimensions, particularly in the height of the slot, which can influence torque expression [4].

Achieving a good aesthetic outcome is highly dependent on successful expression of the torque prescriptions on the anterior teeth [5], and oversized bracket slots can cause an unexpected loss of torque of the anterior teeth, particularly when closing buccal spaces with a pre-adjusted edgewise appliance [6].

There have been several studies that investigated the accuracy of orthodontic bracket slots. Kusy and Whitley [7] analyzed the accuracy of 24 stainless steel, titanium, and ceramic bracket slot sizes. They reached the conclusion that three bracket slots were smaller than what was specified by the manufacturers, and 20 others were larger than specified. Cash et al. [8] investigated the slot size and geometry of 11 different commercially available brackets, some of which were ceramic brackets. The authors measured the dimensions at the base of the slot and at the top of the slot. They reached the conclusion that all brackets examined were oversized by between 5% and 24%. Albertini et al. [9] evaluated the play and torque expression of self-ligating and conventionally ligated lingual brackets and found that the type of ligation had no effect on torque expression in lingual orthodontics. Differences in third-order force expression amongst the systems studied were caused by the dimensional accuracy of arch-wires and slots.

Despite its significance, orthodontic bracket manufacturers rarely divulge the tolerance of bracket slot dimensions [10]. Therefore, it is important that studies are undertaken to ascertain the accuracy of bracket slot dimensions, both from the same manufacturers and between different manufacturers, to ensure predictability in orthodontic treatment outcomes. There is a scarcity of literature focusing on ceramic orthodontic brackets, and the brackets that have been included in previous studies are largely no longer available. Therefore, the aim of this study was to determine the accuracy and precision of the bracket slot height—at the face of the slot (external height) and at the base of the slot (internal height)—in some of the most popular commercially available ceramic bracket systems with the McLaughlin–Bennett–Trevisi (MBT) prescription and 0.022″ slot. Whether the slot remains constant from the slot entrance to the base was also to be determined.

## 2. Materials and Methods

**Figure 1:**
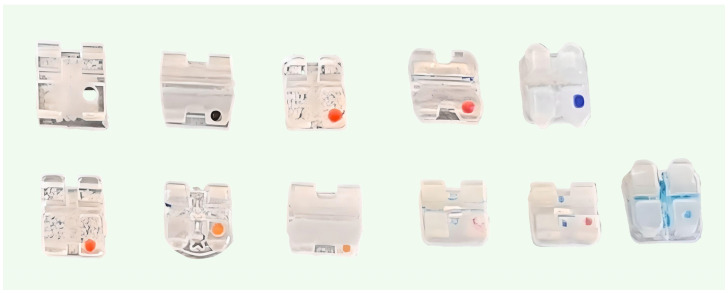
The brackets used in the study, from top left: American Orthodontics Radiance Plus^®^ and 20/40 Bracket^®^; DB Orthodontics Spa^®^; Forestadent Glam^®^; Ormco Symetri^®^; OrthoCare Purity^®^; TOC Ghost Advanced^®^ and Verve^®^; TP Orthodontics ClearVu^®^; InVu^®^ and 3M Clarity Advanced^®^.

Eleven brands of ceramic brackets from eight popular manufacturers were purchased (Figure 1), with five upper right central incisor brackets used for accuracy and precision measurements (n = 55): American Orthodontics Radiance Plus^®^ and 20/40 Bracket^®^ (American Orthodontics, Sheboygan, WI, USA); DB Orthodontics Spa^®^ (DB Orthodontics, Silsden, West Yorkshire, UK); Forestadent Glam^®^ (Forestadent Bernhard Förster GmbH, Pforzheim, Germany); Ormco Symetri^®^ (Ormco Corporation, Orange, CA, USA); OrthoCare Purity^®^ (OrthoCare Limited, Saltaire, West Yorkshire, UK); TOC Ghost Advanced^®^ and Verve^®^ (TOC, Avonmouth, Bristol, UK); TP Orthodontics ClearVu^®^ and InVu^®^ (TP Orthodontics Inc., Le Porte, IN, USA); and 3M Clarity Advanced^®^) (3M Unitek, Monrovia, CA, USA). Ethical approval was obtained from the College of Dentistry at King Saud bin Abdulaziz University for Health Sciences (KSAU-HS), King Abdullah International Medical Research Center’s (KAIMRC) (number SP21R/469/12), Riyadh, Saudi Arabia, and the Faculty of Dentistry, BPP University, Birmingham, UK (number BP0171962/250621).

**Figure 2:**
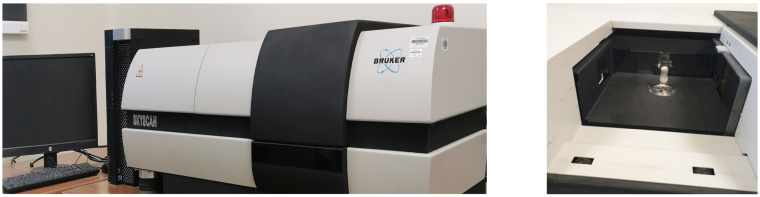
SkyScan 1172 micro-computed tomography (micro-CT) scanner and its scanning chamber that was used in the study.

The brackets were scanned using a micro-computed tomography (Micro-CT) scanner (SkyScan 1172; Bruker microCT, Kontich, Belgium) (Figure 2). The machine has been used previously, given it has a high degree of accuracy of 1 µm [11]. The scanning settings were standardized as follows: 70 kV source voltage, 139 uA source current, 5.9 image pixel size, 0.5 mm aluminum filter, and applied flat-field correction. The micro-CT scanner obtains multiple X-ray ‘shadow’ transmission images of an object from multiple angular views as the object rotates on a high-precision platform. From these ‘shadow’ images, cross-section images of the object are reconstructed in three dimensions (X, Y, and Z) using associated software (NRecon; Bruker microCT, Kontich, Belgium) that utilizes a modified Feldkamp cone-beam algorithm [12]. This creates a complete three-dimensional representation of internal microstructure and density.

**Figure 3:**
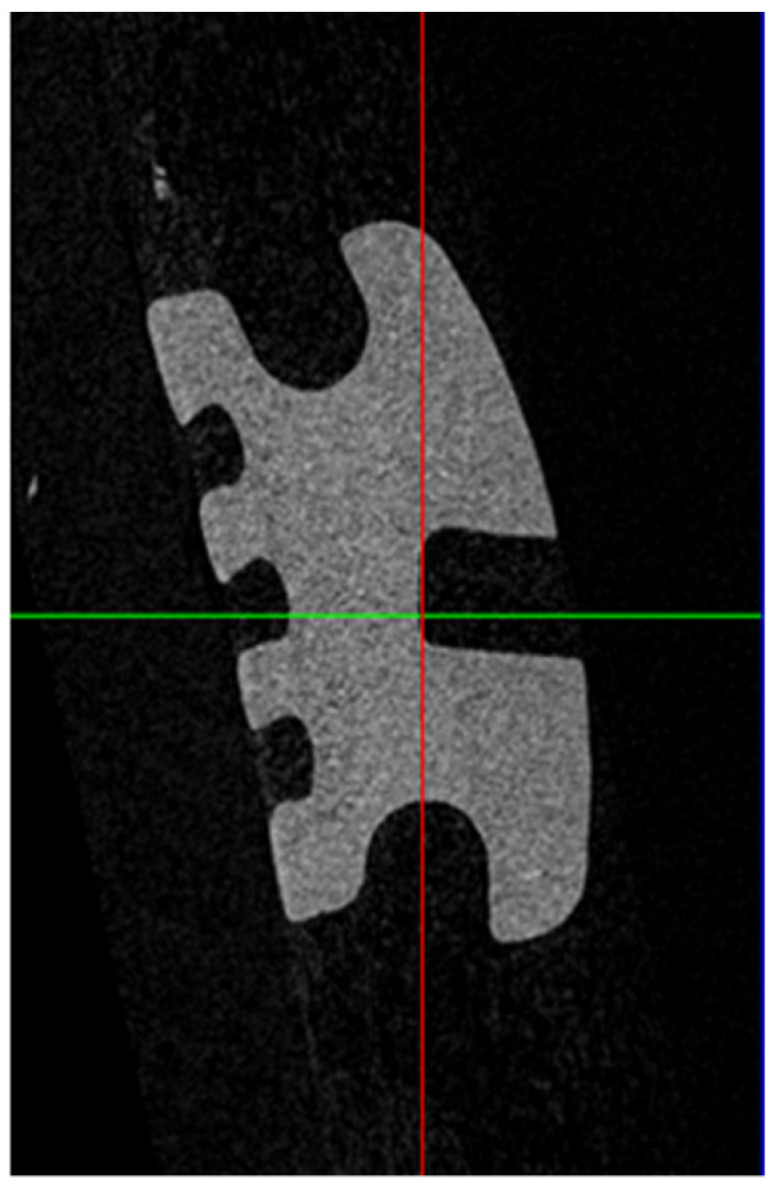
Micro-computed tomography (micro-CT) image of a ceramic bracket.

The resultant 4000 voxel three-dimensional images were manipulated using dedicated software (DataViewer; Bruker microCT, Kontich, Belgium) to standardize the orientation of the image. This was achieved by aligning the base of the bracket slot so that it was parallel to the X–Y plane. Two-dimensional images were obtained in the Y–Z plane at the middle of the distal wing slot on each bracket for analysis. They were then processed using dedicated software (CTAn; Bruker microCT, Kontich, Belgium), where images were binarized by thresholding, and, then, saved for further analysis. The images were exported into image-analysis software (Image J; WS Rasband, US National Institutes of Health, MA, USA), so that measurements could be taken (Figure 3).

**Figure 4:**
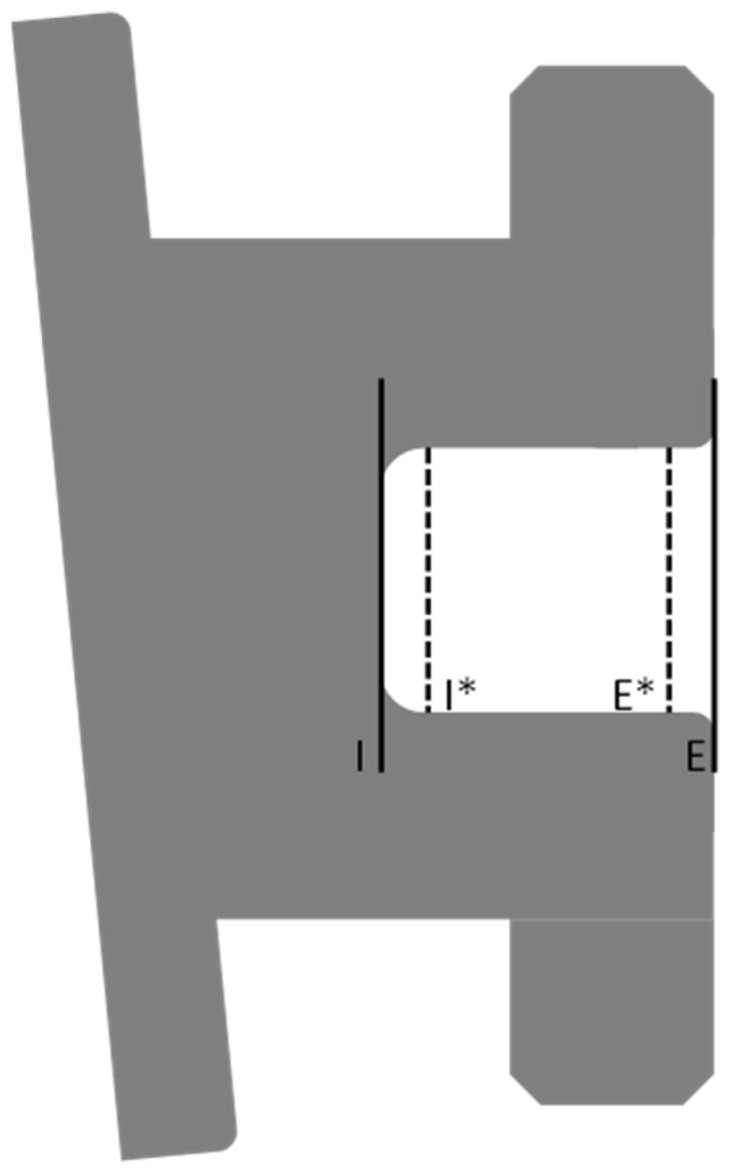
Bracket slot height measurements. The middle of the distal wing slot was selected, and measurements were taken perpendicular to the slot base. From the base of the slot (internal slot height) (I), a distance of 100 µm was taken to determine the internal slot height measurement (I*). From the slot opening (external slot height) (E), a distance of 100 µm was taken to determine the external slot height measurement (E*).

Due to the rounding of bracket slots at their lateral edges, the middle of the distal wing slot on each bracket was selected for measurement. The measurement was taken perpendicular to the slot base. Similarly, since slots can be rounded at the slot entrance and base, the bracket slot height 100 µm from the slot entrance (external height) and 100 µm from the slot base (internal height) were measured (Figure 4). This enabled the evaluation of slot size accuracy as well as whether the height was constant from the entrance of the slot to the base. This measurement method is consistent with the method used in other studies [13,14]. All measurements were rounded to the nearest micron and then, converted to 1/1000th of an inch. The micro-CT scanning and measurements were performed by an experienced investigator (M.A.A).

Statistical analysis was performed using SPSS (Statistical Package for Social Sciences) for Windows version 25.0 (IBM SPSS Inc., Chicago, IL, USA). The data were found to be normally distributed (Kolmogorov–Smirnova (*p =* 0.2) and Shapiro–Wilk (*p =* 0.42)). A One-Sample *t*-Test was used to compare the actual slot dimensions with expected dimensions. A paired *t*-Test was used to compare the mean external and internal heights for each bracket brand to determine the parallelism of the bracket slot for each brand. A one-way ANOVA test followed by Tukey’s post hoc analysis was used to compare the mean of external and internal slot height among groups of manufacturers against the standard marketed size of 0.022. The level of significance was set at *p* ≤ 0.05.

Repeatability testing was undertaken by remeasuring the slot heights of 25% of the samples after three weeks. Validity of the measurement protocol was assessed using the intraclass correlation coefficient (ICC), and excellent reliability was observed with ICC values between 0.950 and 0.99.

## 3. Results

The minimum and maximum registered values for external and internal heights of each bracket brand are presented in Table 1, along with the mean difference to the dimension expected (0.022″). The lowest external height was seen in OrthoCare Purity^®^ at 0.02264″ (2.9%), and the greatest in TOC Ghost Advanced^®^ at 0.02736″ (24.4%). The lowest internal height was seen in Forestadent Glam^®^ at 0.02020″ (−8.2%), and the greatest in TOC Ghost Advanced^®^ at 0.02547″ (15.8%).

OrthoCare Purity^®^ measurements corresponded mostly closely with the expected measurements, with a mean external height difference of 0.000949″ and internal height difference of 0.000458″ from the expected value (0.022″) (Table 1). TOC Ghost Advanced^®^ had the greatest differences, with a mean external height difference of 0.004582″ and mean internal height difference of 0.002303″.

When the range for the measurements of each of the five brackets per brand was tested (Table 1), TP InVu^®^ was found to be the most precise bracket for external height (range = 0.00043″) and American Orthodontics 20/40^®^ for internal height (range = 0.00028″). Ormco Symetri^®^ was the least precise for external height (range = 0.00181″) and TOC Ghost Advanced^®^ for internal height (range = 0.00256″).

There was a statistically significant difference between the slot dimensions recorded in the study compared to the expected values for all brands (*p* ≤ 0.05). Only the internal height was found to be non-significant for DB Orthodontics Spa^®^ (Table 1).

**Figure 5:**
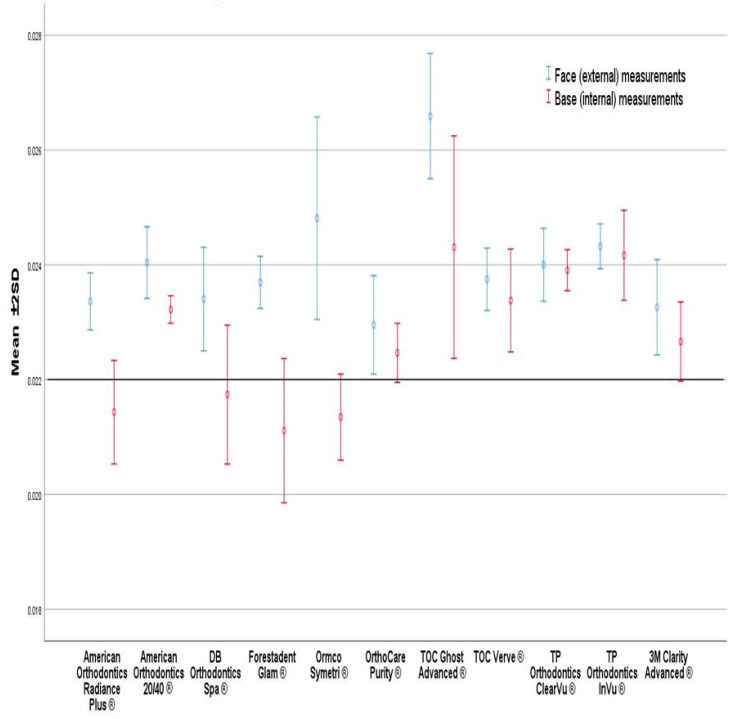
Face (external) and base (internal) measurements means for each bracket type.

The mean external and internal height for each bracket brand is presented in Figure 5 and Table 2. The brand with an external height closest to the expected value of 0.022″ was the OrthoCare Purity^®^ with a mean external height of 0.02296″ (4.4%) and the bracket that was furthest from the expected value was TOC Ghost Advanced^®^ at 0.02659″ (20.9%). The internal height that was closest to the expected value was at 0.02175″ (−1.1%) for DB Orthodontics Spa^®^ and the greatest difference was for TOC Ghost Advanced^®^ at 0.02431″ (10.5%). All brackets had a statistically significant difference between external and internal heights (*p* ≤ 0.05) except for TOC Verve^®^, TP Orthodontics ClearVu^®^ and InVu^®^, and 3M Clarity Advanced^®^.

**Figure 6:**
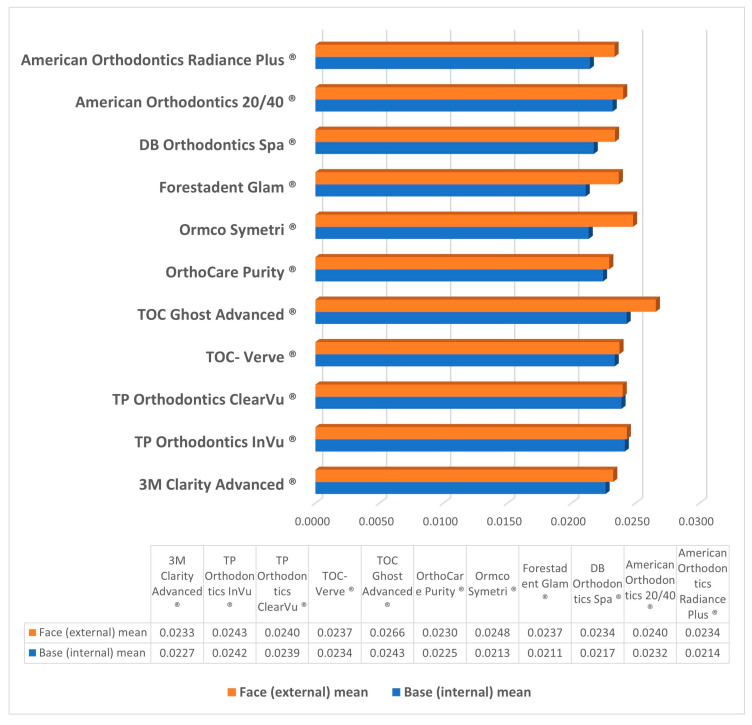
Mean face (external) slot height and base (internal) slot height for each of the 11 branded brackets. Values are displayed in 1/1000th of an inch.

**Figure 7:**
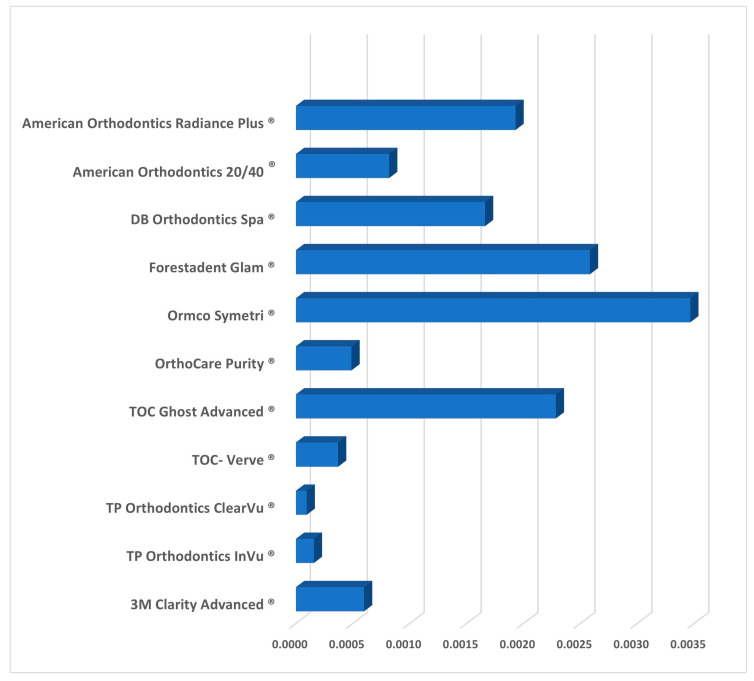
The difference between the face (external) mean and the base (internal) mean for each group.

External and internal measurements means for each bracket type were plotted using error bars with ±2 SD. This shows the variations between the different groups. While the majority of groups are above the specified 0.022″ measurement line, four groups fall beneath that (Figure 5). The mean external and internal heights for each bracket brand are presented in Figure 6 and Figure 7, and Table 2.

In assessing slot geometry, all brackets demonstrated a higher mean external slot height compared to the internal measurements at the base of the bracket. Therefore, all brackets in this study presented with divergent slots (Table 2, Figure 6 and Figure 7). TP Orthodontics ClearVu^®^ showed the most parallelism with a mean difference of 0.000094″ between external and internal heights, and Ormco Symetri^®^ the least with a change of 0.003465″ between external and internal heights. However, the bracket that exhibited the most accurate dimensions for the internal slot height was OrthoCare Purity^®^ with a mean external height of 0.02296″ and a mean internal height of 0.02247″ and a mean difference of 0.000488″.

A one-way ANOVA test and Tukey’s honest significant difference (HSD) post hoc analysis were carried out for both measurements from the external side (Table 3) of the slots and the internal side (Table 4). This analysis compares the means of each group and is used to assess the significance of differences between group means. This was conducted after adding a 12th group with the standard declared measurements of the brackets (0.022″). In the external side, there are six homogenous subsets. The declared measurement differs significantly from all 11 groups when comparing the measurements of the slot from the external side (Table 3). However, the case is different when comparing the measurements of the slot from the internal side. In that case, six out of the 11 groups (namely, Forestadent Glam^®^, Ormco Symetri^®^, American Orthodontics Radiance Plus^®^, DB Orthodontics Spa^®^, OrthoCare Purity^®^, and 3M Clarity Advanced^®^) did not differ significantly from the standard 0.22″ (Table 4), although, three of them (subset 1) differ from the other two (subset 2) significantly. Of the internal means, there are only five homogenous subsets compared to six subsets in the external means.

## 4. Discussion

Tooth positioning in orthodontic treatment is achieved by an effective interaction between the archwire and the bracket slot and its built-in prescription. For this prescription to be expressed successfully, resulting in correct three-dimensional tooth positioning, the fit between the archwire and the bracket slot must be as intimate as possible [10].

Although large discrepancies in the accuracy of the bracket slots can influence all three movements incorporated in the bracket slot prescriptions, torque expression—the buccolingual inclination of the teeth—is most affected [15], with even small discrepancies between the expected dimensions of the bracket slot and the actual dimensions, having a significant impact on the effectiveness of torque expression [16].

Alexander [17] has described that for every 0.001″ of play between the archwire and the bracket slot, 5° of effective torque is lost, assuming the walls are completely parallel and smooth. A significant amount of extra torque would need to be added to the current prescriptions to overcome the increased play between the archwire and bracket slot.

This was the first study undertaken to evaluate accuracy and precision of slot dimensions in a range of regular ligation ceramic brackets. In this study, all bracket systems analyzed revealed an oversized external height of the bracket on average 9.2% (mean range 4.4% to 20.9%) greater than the expected value, which is consistent with previous studies that found the external height to be 8% [8], and 10% [12], greater than expected. Similarly, the internal height was 3.2% (mean range −4.1 to 10.5%) greater than the expected value on average; however, some bracket slots were undersized, and others were oversized. This has been reported in other studies that have found bracket slots to be undersized in 15% [8], 22.3% [18], and 36% [13] of all brackets analyzed. None of the bracket brands included in this study had dimensions that were within the dimensional tolerances of 0.0004-inch defined by the ISO 27020:2019 [15]. This mismatch is comparable to the findings of Divya et al., who revealed statistically significant dimension discrepancies between the declared and measured values of brackets [19].

All 11 different bracket brands analyzed exhibited divergent walls, with the external height of the bracket slot higher than the internal height at the internal (base) of the bracket. These results are in accordance with most studies that analyzed slot parallelism. Lee et al. [20] also found that all bracket brands analyzed in their study had divergent walls, while Lefebvre et al. [14] reported divergent slots in 85% of the brackets analyzed. Similarly, bracket slots featured divergent walls in nine of the 12 brands analyzed by Martínez et al. [21]; however, Cash et al. [8] found parallel slots in four of the 11 brackets tested, convergent slots in five, and divergent slots in only two of the bracket brands.

The results of the present study indicate that due to the lack of dimensional accuracy of the bracket systems included in this study, although it is difficult to predict the actual amount of play between the archwire and the bracket slot as most of the brackets presented with oversized slots, the orthodontist should assume that the actual play between the archwire and the bracket slot is most likely higher than expected. Clinically, this is unacceptable, as the torque moments that would otherwise be achieved by the same archwire and bracket slot combinations will be significantly reduced.

If we consider the results obtained in this study, for example, for the 3M Clarity Advanced^®^ bracket, which registered a mean difference to the expected value of 0.001327″ (external height) and 0.00067″ (internal height), assuming a full-size archwire is used with the same height as the expected value of the bracket slot height, around 5° of effective torque would be lost. If we consider that the working archwire used would be a 0.019″ by 0.025″, a play of 0.003″ results in an extra 15° of torque loss, totaling 20° overall. To achieve the torque moments intended for a maxillary central incisor bracket with an MBT prescription, the in-built torque prescription would need to be increased to 37°.

There are other factors that can influence torque expression, such as the accuracy of the archwire dimensions. Significant discrepancies between the expected archwire values and their actual dimensions have previously been reported [16]. In addition, the exact geometry of the archwires can also influence torque expression, as beveled edges can affect the engagement between the archwire and the slot, thus, compromising the force couple necessary for appropriate transmission of the built-in torque prescription.

A limitation of the present study is that bracket slots were measured on the distal wing only, and no measurements were taken from the mesial side of the brackets. This methodology is well established [8,13,22]; however, measuring the mesial as well as the distal aspect would have allowed comparison of the symmetry of the bracket slots.

## 5. Conclusions

The results obtained in this study indicate that orthodontic bracket slots are larger than expected and slot parallelism was not observed in any bracket brand. Similarly, slot dimensions are imprecise where two ‘identical’ brackets have different slot sizes. The clinician should, therefore, assume that play is most likely higher than expected. Adherence to the recommended dimensional tolerances should be enforced during the manufacturing process of orthodontic brackets to be within the dimensional tolerances of 0.0004-inch specified by the norm for standardization of orthodontic products (ISO 27020:2019).

More work is required to ensure that any new brackets introduced into the market overcome this imprecision. Furthermore, future industry research and development would ensure that novel technology is utilized in the bracket-production process to eradicate this flaw.

## Figures and Tables

**Table 1:** Minimum and maximum registered dimensions for face (external) and base (internal) slot height in each upper right central incisor bracket and mean difference to expected value.

Bracket		Minimum	% Deviation from Expected (0.022″)	Maximum	% Deviation from Expected (0.022″)	Range	Mean Difference to Expected Value	*p*-Value
American Orthodontics Radiance Plus^®^	External.	23.07	4.90%	23.62	7.40%	0.55	1.363	***
	Internal	20.75	−5.7%	21.86	−0.6%	1.11	−0.565	*
American Orthodontics 20/40^®^	External.	23.62	7.40%	24.49	11.30%	0.87	2.043	***
	Internal	23.11	5.10%	23.39	6.30%	0.28	1.223	***
DB Orthodontics Spa^®^	External.	23.03	4.70%	24.09	9.50%	1.06	1.404	**
	Internal	21.14	−3.9%	22.6	2.70%	1.46	−0.262	0.391
Forestadent Glam^®^	External.	23.54	7%	24.09	9.50%	0.55	1.699	***
	Internal	20.2	−8.2%	21.97	−0.1%	1.77	−0.894	*
Ormco Symetri^®^	External.	23.94	8.80%	25.75	17.10%	1.81	2.815	**
	Internal	20.79	−5.5%	21.69	−1.4%	0.9	−0.652	*
OrthoCare Purity^®^	External.	22.64	2.90%	23.62	7.40%	0.98	0.949	**
	Internal	22.17	0.80%	22.68	3.10%	0.51	0.458	*
TOC Ghost Advanced^®^	External.	25.94	17.90%	27.36	24.40%	1.42	4.582	***
	Internal	22.91	4.10%	25.47	15.80%	2.56	2.303	**
TOC Verve^®^	External.	23.46	6.60%	24.09	9.50%	0.63	1.747	***
	Internal	23.03	4.70%	24.06	9.30%	1.03	1.379	**
TP	External.	23.62	7.40%	24.49	11.30%	0.87	2.003	***
Orthodontics ClearVu^®^	Internal	23.7	7.70%	24.09	9.50%	0.39	1.901	***
TP	External.	24.06	9.40%	24.49	11.30%	0.43	2.326	***
Orthodontics InVu^®^	Internal	23.62	7.40%	24.57	11.70%	0.95	2.169	***
3M Clarity Advanced^®^	External.	22.68	3.10%	23.7	7.70%	1.02	1.263	**
	Internal	22.2	0.90%	23.15	5.20%	0.95	0.664	*

All values given in 1/1000th of an inch. ‘External’ refers to external (face) slot height and ‘Internal’ refers to internal (base) slot height. (*** *p* ≤ 0.001; ** *p* ≤ 0.01; * *p* ≤ 0.05) using One-Sample *t*-Test.

**Table 2:** Mean face (external) and base (internal) slot dimensions of the upper right central incisor bracket and mean difference between the face (external) and internal slot height given in 1/1000th of an inch.

Bracket	External Height			% Deviation from Expected (0.022″)	Internal Height			% Deviation from Expected (0.022″)	Mean Difference of External/ Internal Height	*p*-Value
	M	SD	CV %		M	SD	CV%			
American Orthodontics Radiance Plus^®^	23.37	0.251	1.07%	6.30%	21.44	0.248	1.16%	−2.6%	1.929	**
American Orthodontics 20/40^®^	24.05	0.312	1.30%	9.30%	23.23	0.124	0.53%	5.60%	0.819	**
DB Orthodontics Spa^®^	23.41	0.455	1.94%	6.40%	21.75	0.609	2.80%	−1.1%	1.661	***
Forestadent Glam^®^	23.71	0.227	0.96%	7.80%	21.11	0.632	2.99%	−4.1%	2.583	***
Ormco Symetri^®^	24.82	0.884	3.56%	12.80%	21.36	0.153	0.72%	−2.9%	3.465	***
OrthoCare Purity^®^	22.96	0.438	1.91%	4.40%	22.47	0.269	1.20%	2.10%	0.488	**
TOC Ghost Advanced^®^	26.59	0.545	2.05%	20.90%	24.31	0.966	3.97%	10.50%	2.284	**
TOC Verve^®^	23.76	0.271	1.14%	8%	23.39	0.439	1.88%	6.30%	0.370	0.105
TP Orthodontics ClearVu^®^	24.01	0.318	1.32%	9.10%	23.9	0.18	0.75%	8.60%	0.094	0.408
TP Orthodontics InVu^®^	24.33	0.202	0.83%	10.60%	24.17	0.388	1.61%	9.90%	0.158	0.397
3M Clarity Advanced^®^	23.27	0.410	1.76%	5.80%	22.67	0.347	1.53%	3.10%	0.598	0.086

(*** *p ≤* 0.001; ** *p ≤* 0.01) using Paired *t*-Test. M: mean, SD: standard deviation, CV: coefficient of variations.

**Table 3:** Tukey’s honest significant difference analysis (HSDA) for the internal measurements.

Internal Measurement—Tukey’s HSDA						
Group Number	n	Subset for Alpha = 0.05				
		1	2	3	4	5
Forestadent Glam^®^	5	0.0211				
Ormco Symetri^®^	5	0.0213				
American Orthodontics Radiance Plus^®^	5	0.0214				
DB Orthodontics Spa^®^	5	0.0217	0.0217			
Standard 22	5	0.022	0.022			
OrthoCare Purity^®^	5		0.0225	0.0225		
3M Clarity Advanced^®^	5		0.0227	0.0227		
American Orthodontics 20/40	5			0.0232	0.0232	
TOC Verve^®^	5			0.0234	0.0234	0.0234
TP Orthodontics ClearVu^®^	5				0.0239	0.0239
TP Orthodontics InVu^®^	5				0.0242	0.0242
TOC Ghost Advanced^®^	5					0.0243
Sig.		0.138	0.109	0.116	0.091	0.103

Means for groups in homogeneous subsets are displayed. Uses harmonic mean sample size = 5.000.

**Table 4:** Tukey’s honest significant difference analysis (HSDA) for the external measurements.

External Measurement—Tukey’s HSDA							
Group Number	n	Subset for Alpha = 0.05					
		1	2	3	4	5	6
Standard 22	5	0.022					
OrthoCare Purity^®^	5		0.023				
3M Clarity Advanced^®^	5		0.0233	0.0233			
American Orthodontics Radiance Plus^®^	5		0.0234	0.0234			
DB Orthodontics Spa^®^	5		0.0234	0.0234			
Forestadent Glam^®^	5		0.0237	0.0237	0.0237		
TOC Verve^®^	5		0.0237	0.0237	0.0237		
TP Orthodontics ClearVu^®^	5			0.024	0.024	0.024	
American Orthodontics 20/40	5			0.024	0.024	0.024	
TP Orthodontics InVu^®^	5				0.0243	0.0243	
Ormco Symetri^®^	5					0.0248	
TOC Ghost Advanced^®^	5						0.0266
Sig.		1	0.1301	0.1483	0.4235	0.1137	1

Means for groups in homogeneous subsets are displayed. Uses harmonic mean sample size = 5.000.

## Data Availability

Raw data are available upon request from the authors.
